# Supplementary material for: High Triglyceride-Glucose Index with Renal Hyperfiltration and Albuminuria in Young Adults: The Korea National Health and Nutrition Examination Survey (KNHANES V, VI, and VIII)
Source: J Clin Med. 2022 Oct 29;11(21):6419. doi: 10.3390/jcm11216419 (PMC9655420; doi:10.3390/jcm11216419)
Supplement: Supplementary file 1 [file jcm-11-06419-s001.zip › jcm-1972327-Supplementary.pdf]

Online-Only Supplementary Materials

High Triglyceride-Glucose Index with Renal Hyperfiltration and Albuminuria in Young Adults: the Korea National Health and Nutrition Examination Survey (KNHANES V, VI, and VIII)

Donghwan Oh [1,2], Sang Ho Park [1], Seoyoung Lee [1,2], Eunji Yang [1,2], Hoon Young Choi [1,2,3], Hyeong Cheon Park [1,2,3] and Jong Hyun Jhee [1,2]

[1] *Division of Nephrology, Department of Internal Medicine, Gangnam Severance Hospital, Seoul, Korea*

[2] *Department of Internal Medicine, Yonsei University College of Medicine, Seoul, Korea*

[3] *Severance Institute for Vascular and Metabolic Research, Yonsei University College of Medicine, Seoul, Korea*

Corresponding Author:

Jong Hyun Jhee, M.D., Ph.D.

Division of Nephrology, Department of Internal Medicine, Gangnam Severance Hospital, Yonsei University College of Medicine, 211 Eonju-ro, Gangnam-gu, Seoul 06273, Korea

Phone: +82-2-2019-4368; Fax: +82-2-3463-3882 E-mail: jjhlove77@yuhs.ac

Content list:

Supplementary Methods

Supplementary Figure S1. ROC curve analyses for the risk of albuminuria with TyG index with or without RHF

Supplementary Table S1. Multi-collinearity evaluation through the tolerance and VIF values of diabetes, smoking, and BMI

Supplementary Table S2. Interaction analysis of association between TyG index and albuminuria according to the presence of diabetes, status of smoking, and BMI

Supplementary Table S3. Interaction analysis of the association between TyG index and RHF according to the presence of diabetes, status of smoking, and BMI

Supplementary Table S4. Sensitivity analysis of the combination of TyG index and RHF, with the risk of albuminuria, according to the glucose level

Supplementary Table S5. Logistic regression analyses for RHF (95th percentile) according to TyG index

Supplementary Table S6. Association between albuminuria and TyG index with or without RHF (95th percentile)

Supplementary Methods

Anthropometric parameters such as height, body weight, and body mass index (BMI) were measured by trained healthcare providers following the standardized protocols. Smoking and alcohol status were assessed using the survey questionnaires, and the participants were classified into two groups (current or former smoker/drinker vs. never smoker/drinker). Education level was divided into two groups (less than the middle school to middle school graduate vs. high school graduate or higher). Income levels were divided into two groups based on the 50th percentile personal income of the study group each year (low income vs. high income). Blood pressure (BP) was measured following the KNHANES protocol using a sphygmomanometer (Baunmanometer; WA Baum Co., New York, NY, USA) with participants' right arm after resting for 5 min and averaged by three measurements. Hypertension was defined as systolic BP (SBP) ≥140 mmHg or diastolic BP (DBP) ≥90 mmHg, or taking antihypertensive drugs, or previous diagnosis of hypertension by a medical doctor. Diabetes was defined as a glycated hemoglobin (HbA1c) level ≥6.5% or a fasting glucose level ≥126 mg/dL, or using antidiabetic drugs, or previous diagnosis of diabetes by a medical doctor. Participants were defined to have dyslipidemia if they were taking antidyslipidemic drugs or had been diagnosed with dyslipidemia by a medical doctor.

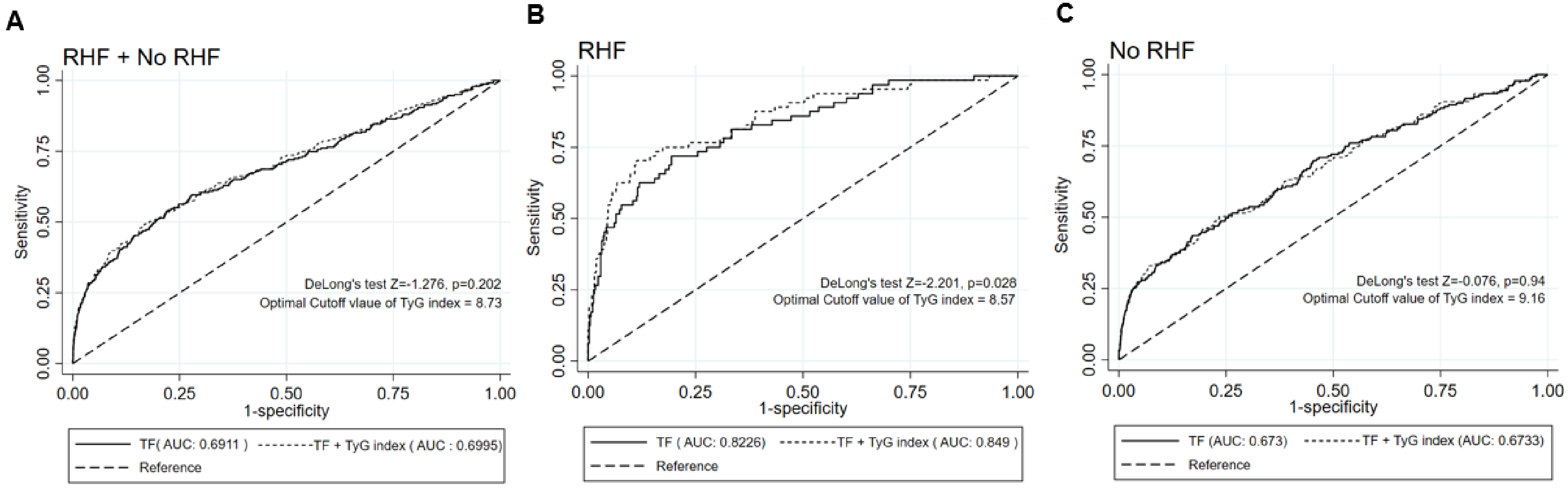

Supplementary Figure S1. ROC curve analyses for the risk of abluminuria with TyG index with or without RHF
Adjusted for age, sex, smoking and alcohol status, education and income levels, BMI, SBP, hemoglobin, eGFR(CKD-EPI), and past history of HTN, DM, and dyslipidemia.

Abbreviations: ROC, receiver operating characteristic; RHF, renal hyperfiltration; TyG index, triglyceride-glucose index; AUC, area under curve; BMI, body mass index; SBP, systolic blood pressure; eGFR, estimated glomerular filtration rate; CKD-EPI, CKD Epidemiology Collaboration; HTN, hypertension; DM, diabetes mellitus

Supplementary Table S1. Multi-collinearity evaluation through the tolerance and VIF values of diabetes, smoking, and BMI.

Variables	VIF	Tolerance
TyG index	1.40	0.7127
Diabetes	1.08	0.9284
Smoking	1.10	0.9094
BMI	1.28	0.7834

Supplementary Table S2. Interaction analysis of association between TyG index and albuminuria according to the presence of diabetes, status of smoking, and BMI.

Variable	Prevalence, n/total (%)	P for interaction	Model 1		Model 2		Model 3*	
			OR (95% CI)	*P*	OR (95% CI)	*P*	OR (95% CI)	*P*
**Diabetes**		0.005						
No	196/5,301 (3.7)		1.73 (1.40-2.12)	<0.001	1.88 (1.50-2.37)	<0.001	1.38 (1.07-1.78)	0.01
Yes	37/119 (31.1)		3.84 (2.05-7.20)	<0.001	3.92 (2.08-7.37)	<0.001	2.94 (1.40-6.18)	0.004
**Smoking**		<0.001						
No	126/3,175 (4.0)		1.86 (1.42-2.43)	<0.001	1.98 (1.49-2.62)	<0.001	1.23 (0.89-1.70)	0.21
Yes	107/2,245 (4.8)		3.31 (2.57-4.26)	<0.001	3.40 (2.61-4.42)	<0.001	1.97 (1.45-2.68)	<0.001
**BMI**		0.003						
<25	133/3,930 (3.4)		1.61 (1.22-2.11)	0.001	1.76 (1.31-2.38)	<0.001	1.30 (0.94-1.79)	0.12
≥25	100/1,490 (6.7)		3.39 (2.54-4.52)	<0.001	3.54 (2.62-4.76)	<0.001	2.16 (1.52-3.06)	<0.001

Note: * Model 3 was adjusted with the covariates excluding the variables corresponding to each subgroup.

Model 1: Unadjusted model

Model 2: Adjusted for age and sex

Model 3: Adjusted for age, sex, smoking and alcohol status, education and income levels, BMI, SBP, hemoglobin, eGFR(CKD-EPI), and past history of HTN, DM, and dyslipidemia

Abbreviations: TyG, triglyceride-glucose; OR, odds ratio; CI, confidence interval; BMI, body mass index; SBP, Systolic blood pressure; eGFR, estimated glomerular filtration rate; CKD-EPI, CKD Epidemiology Collaboration; HTN, hypertension; DM, diabetes mellitus

Supplementary Table S3. Interaction analysis of the association between TyG index and RHF according to the presence of diabetes, status of smoking, and BMI.

Variable	Prevalence, n/total (%)	P for interaction	Model 1		Model 2		Model 3*	
			OR (95% CI)	*P*	OR (95% CI)	*P*	OR (95% CI)	*P*
**Diabetes**		0.96						
No	509/5,301 (9.6)		1.44 (0.88-2.36)	0.15	1.27 (0.76-2.12)	0.37	1.26 (0.69-2.29)	0.45
Yes	34/119 (28.6)		1.97 (1.72-2.26)	<0.001	1.38 (1.16-1.61)	<0.001	1.67 (1.41-1.98)	<0.001
**Smoking**		0.12						
No	226/3,175 (7.1)		1.81 (1.47-2.23)	<0.001	1.37 (1.08-1.72)	0.008	1.53 (1.17-1.99)	0.002
Yes	317/2,245 (14.1)		1.85 (1.56-2.18)	<0.001	1.59 (1.33-1.90)	<0.001	1.68 (1.36-2.07)	<0.001
**BMI**		0.71						
<25	360/3,930 (9.2)		1.94 (1.55-2.42)	<0.001	1.73 (1.37-2.19)	<0.001	1.51 (1.16-1.96)	0.002
≥25	183/1,490 (12.3)		2.22 (1.87-2.64)	<0.001	1.50 (1.23-1.82)	<0.001	1.77 (1.43-2.19)	<0.001

Note: * Model 3 was adjusted with the covariates excluding the variables corresponding to each subgroup.

Model 1: Unadjusted model

Model 2: Adjusted for age and sex

Model 3: Adjusted for age, sex, smoking and alcohol status, education and income levels, BMI, SBP, hemoglobin, and past history of HTN, DM, and dyslipidemia

Abbreviations: TyG, triglyceride-glucose; OR, odds ratio; CI, confidence interval; BMI, body mass index; SBP, Systolic blood pressure; HTN, hypertension; DM, diabetes mellitus

Supplementary Table S4. Sensitivity analysis of the combination of TyG index and RHF, with the risk of albuminuria, according to the glucose level.

Variable		Prevalence* n (%)	Model 1		Model 2		Model 3	
			OR (95% CI)	*P*	OR (95% CI)	*P*	OR (95% CI)	*P*
**Glucose level**								
Prediabetes	**TyG index** (per 1.0 increase) + **RHF**	22 (14.9)	2.58 (1.65-4.04)	<0.001	2.99 (1.86-4.83)	<0.001	2.05 (1.16-3.65)	0.01
Diabetes		19 (55.9)	5.93 (2.16-16.3)	0.001	6.02 (2.12-17.04)	0.001	5.32 (1.34-20.75)	0.02
**Glucose level**								
Prediabetes	**TyG index** (per 1.0 increase) + **no RHF**	38 (3.1)	1.79 (0.94-3.40)	0.08	1.89 (0.97-3.70)	0.06	1.64 (0.72-3.72)	0.24
Diabetes		18 (21.2)	2.11 (0.93-4.81)	0.08	2.24 (0.93-5.38)	0.07	3.00 (0.63-14.25)	0.17

Note: **P* <0.001

Model 1: Unadjusted model

Model 2: Adjusted for age and sex

Model 3: Adjusted for age, sex, smoking and alcohol status, education and income levels, BMI, SBP, hemoglobin, eGFR(CKD-EPI), and past history of HTN, DM, and dyslipidemia

Abbreviations: TyG, triglyceride-glucose; RHF, renal hyperfiltration; OR, odds ratio; CI, confidence interval; BMI, body mass index; SBP, Systolic blood pressure; eGFR, estimated glomerular filtration rate; CKD-EPI, CKD- Epidemiology Collaboration; HTN, hypertension; DM, diabetes mellitus

Supplementary Table S5. Logistic regression analyses for RHF (95th percentile) according to TyG index.

Variable	Prevalence* n (%)	Model 1		Model 2		Model 3	
		OR (95% CI)	*P*	OR (95% CI)	*P*	OR (95% CI)	*P*
**TyG index** (per 1.0 increase)	271 (5.0)	2.30 (1.95-2.70)	<0.001	1.85 (1.54-2.22)	<0.001	2.08 (1.68-2.58)	<0.001
**Tertile of TyG index**							
Tertile 1 (lowest)	49 (2.7)			(Reference)			
Tertile 2	69 (3.8)	1.43 (0.99-2.07)	0.06	1.12 (0.77-1.64)	0.56	1.21 (0.82-1.78)	0.33
Tertile 3 (highest)	153 (8.5)	3.32 (2.39-4.61)	<0.001	2.08 (1.46-2.97)	<0.001	2.44 (1.66-3.60)	<0.001

Note: **P* <0.001,

Model 1: Unadjusted model

Model 2: Adjusted for age and sex

Model 3: Adjusted for age, sex, smoking and alcohol status, education and income levels, BMI, SBP, hemoglobin, albuminuria, and past history of HTN, DM, and dyslipidemia

Abbreviations: RHF, renal hyperfiltration; TyG, triglyceride-glucose; OR, odds ratio; CI, confidence interval; BMI, body mass index; SBP, Systolic blood pressure; HTN, hypertension; DM, diabetes mellitus

Supplementary Table S6. Association between albuminuria and TyG index with or without RHF (95th percentile).

Variable	Prevalence* n (%)	Model 1		Model 2		Model 3	
		OR (95% CI)	*P*	OR (95% CI)	*P*	OR (95% CI)	*P*
**With RHF**							
**TyG index** (per 1.0 increase)	38 (14.0)	4.07 (2.47-6.73)	<0.001	3.87 (2.32-6.45)	<0.001	2.04 (1.06-3.91)	0.03
**Tertile of TyG index**							
Tertile 1 (lowest, n=91)	3 (3.3)			(reference)			
Tertile 2 (n=90)	9 (10.0)	3.26 (0.85-12.46)	0.08	3.03 (0.78-11.69)	0.11	2.24 (0.52-9.60)	0.28
Tertile 3 (highest, n=90)	26 (28.9)	11.92 (3.46-41.08)	<0.001	10.39 (2.97-36.34)	<0.001	3.55 (1.65-15.11)	0.04
**Without RHF**							
**TyG index** (per 1.0 increase)	195 (3.8)	2.00 (1.65-2.43)	<0.001	2.28 (1.84-2.82)	<0.001	1.43 (1.11-1.85)	0.06
**Tertile of TyG index**							
Tertile 1 (lowest, n=1,720)	43 (2.5)			(reference)			
Tertile 2 (n=1,714)	57 (3.3)	1.34 (0.90-2.01)	0.15	1.44 (0.96-2.17)	0.08	1.30 (0.86-1.96)	0.22
Tertile 3 (highest, n=1,715)	95 (5.5)	2.29 (1.59-3.30)	<0.001	2.66 (1.79-3.95)	<0.001	1.47 (0.94-2.30)	0.10

Note: **P* <0.001

Model 1: Unadjusted model

Model 2: Adjusted for age and sex

Model 3: Adjusted for age, sex, smoking and alcohol status, education and income levels, BMI, SBP, hemoglobin, eGFR(CKD-EPI), and past history of HTN, DM, and dyslipidemia

Abbreviations: CKD, chronic kidney disease; RHF, renal hyperfiltration; TyG, triglyceride-glucose; OR, odds ratio; CI, confidence interval; BMI, body mass index; SBP, Systolic blood pressure; eGFR, estimated glomerular filtration rate; CKD-EPI, CKD Epidemiology Collaboration; HTN, hypertension; DM, diabetes mellitus
